# Massively parallel sequencing of *Cannabis sativa* chloroplast hotspots for forensic typing

**DOI:** 10.1186/s42238-022-00123-2

**Published:** 2022-03-17

**Authors:** Madeline G. Roman, Ryan Gutierrez, Rachel Houston

**Affiliations:** Department of Forensic Science, Sam Houston State University, 1003 Bowers Blvd, Huntsville, TX 77340 USA

**Keywords:** *Cannabis sativa*, Massively parallel sequencing, Chloroplast DNA, Genetic assay

## Abstract

**Background:**

Marijuana (*Cannabis sativa*) is the most commonly used illicit drug in the USA, and the use of DNA barcodes could assist drug trafficking investigations by indicating the biogeographical origin and crop type of a sample and providing a means for linking cases. Additionally, the legality of marijuana in the USA remains complicated with some states fully legalizing marijuana for recreational use while federally marijuana remains completely illegal. Massively parallel sequencing (MPS) offers distinct advantages over capillary electrophoresis (CE), including more comprehensive coverage of target loci, analysis of hundreds of markers simultaneously, and high throughput capabilities.

**Methods:**

This study reports on the development of a MiSeq FGx® assay targeting seven “hotspot” regions in the *Cannabis sativa* chloroplast genome that are highly polymorphic and informative in attempts to determine biogeographical origin and distinguishing between marijuana and hemp. Sequencing results were compared to previous studies that used CE-based genotyping methods.

**Results:**

A total of 49 polymorphisms were observed, 16 of which have not been previously reported. Additionally, sequence data revealed isoalleles at one locus, which were able to differentiate two samples that had the same haplotype using CE-based methods. This study reports preliminary results from sequencing 14 hemp and marijuana samples from different countries using the developed MPS assay.

**Conclusion:**

Future studies should genotype a more comprehensive sample set from around the world to build a haplotype database, which could be used to provide investigative leads for law enforcement agencies investigating marijuana trafficking.

**Supplementary Information:**

The online version contains supplementary material available at 10.1186/s42238-022-00123-2.

## Background

Massively parallel sequencing (MPS), also called next generation sequencing (NGS), is a high throughput technique capable of collecting DNA sequence data from multiple targets and multiple samples in parallel. It offers several distinct advantages over traditional DNA typing using capillary electrophoresis (CE), including providing more comprehensive coverage of target markers (sequence data in addition to length) and the ability to analyze hundreds or thousands of targets at a time (Bruijns et al. 2018; Moorthie et al. 2011), compared to only about 25 loci by CE for a five-dye short tandem repeat (STR) kit (Lazaruk et al. 1998). Sequence data may elucidate isoalleles, alleles which have the same length and appear identical on CE but actually have different sequences, leading to more discriminatory results. Isoalleles may differ in their repeat structure or contain variants in the flanking regions, and the International Society of Forensic Genetics (ISFG) has reported guidelines to standardize the nomenclature for these sequence variable alleles (Parson et al. 2016). Costs and run times associated with MPS have dropped substantially, making targeted MPS assays a cost-effective approach for characterizing samples for genetic individualization or identification (Moorthie et al. 2011).

In forensics, MPS assays have been used for human identification purposes, including sequencing autosomal STRs and single nucleotide polymorphisms (SNPs) (Eduardoff et al. 2015; Guo et al. 2017; Kim et al. 2016; Seo et al. 2013; Wang et al. 2017), mitochondrial DNA analysis (Davis et al. 2015), phenotype prediction (Mehta et al. 2016), and other purposes (Bruijns et al. 2018; Budowle et al. 2017). While much of the forensic research on MPS has focused on human DNA, its use for forensic plant science has recently been investigated (Houston et al., 2018a, b). Houston et al. reported an MPS panel for the Ion S5™ consisting of twelve autosomal STRs in *Cannabis sativa* (marijuana). Results showed concordance with CE methods, and isoalleles were found at eight of the loci, providing a higher discriminatory power compared to CE (Houston et al., 2018a, b). MPS has been also been used for DNA barcoding studies in animals and has shown better recovery, reduced costs, and faster processing times compared to traditional Sanger sequencing (Shokralla et al. 2015). Studies involving the use of MPS for DNA barcoding in plants have been limited but do show the advantage of simultaneous analysis of multiple barcodes for enhanced phylogenetic resolution (Parks et al. 2009; Sucher et al. 2012). Recent studies reported chloroplast DNA barcoding markers in *C. sativa* that were informative for biogeographical origin and crop type prediction (Roman et al. 2019; Roman and Houston 2020). Since these regions represent the most highly polymorphic regions of the *C. sativa* chloroplast genome, they are referred to as “hotspots.” The polymorphisms were genotyped using CE-based methods, and Sanger sequencing revealed isoalleles at several loci with different repeat sequences or variations in the flanking regions that were not detected by CE (Roman et al. 2019; Roman and Houston 2020). This study seeks to expand upon previous studies by incorporating the “hotspot” barcoding regions into an MPS assay to provide more discriminatory results and a high throughput method for building a database of *C. sativa* chloroplast haplotypes.

Full chloroplast genome sequences have been reported for several marijuana and hemp cultivars (Matielo et al. 2020; Oh et al. 2016; Vergara et al. 2016). The full genome is 153,871 bp (Carmagnola and Dagestani cultivars) and contains 83 genes (Vergara et al. 2016). In comparison, the human mitochondrial genome is 16,569 bp (Anderson et al. 1981) (about a tenth of the size), and typically mitochondrial DNA analyses only involve sequencing a portion of the genome, usually the hypervariable regions (HV1 and HV2) (Ingman and Gyllensten 2006; Miller and Budowle 2001). Due to the large size of the *C. sativa* chloroplast genome, sequencing targeted regions (barcoding markers) gives better coverage and increased throughput capabilities compared to whole genome sequencing. The chloroplast genome is AT-rich (63%) and contains numerous homopolymeric stretches (Vergara et al. 2016). The MiSeq FGx® platform was chosen for sequencing in this study because has been shown to have a higher fidelity when sequencing homopolymeric stretches of DNA (Duke et al. 2015; Loman et al. 2012; McElhoe et al. 2014), and many of the polymorphisms identified in the previous studies (Roman et al. 2019; Roman and Houston 2020; Cheng and Houston, 2021) were homopolymeric STRs (hSTRs).

This study seeks to design an MPS panel for the MiSeq FGx® consisting of seven highly polymorphic “hotspot” regions in the *C. sativa* chloroplast (*trnK-matK-trnK*, *rps16*, *trnS-trnG*, *ycf3*, *accD-psaI*, *clpP*, and *rpl32-trnL*) to provide additional sequence data, discover isoalleles, and provide a high throughput method for creating a haplotype DNA database for hemp and marijuana samples. This assay could provide important investigative leads for law enforcement agencies investigating marijuana trafficking into and within the USA.

## Methods

### DNA samples

Hemp samples from Canada were purchased online from Badia Spices Inc. (Doral, FL, USA; N=1 (H2-4)) and Navitas Organics (Novato, CA, USA; *N*=1 (H3-3)). USA hemp samples were purchased from American Hemp Harvest (Boulder, CO, USA; *N*=4 (H5-4, NT H5-1, NT H5-2, NT H5-4)) and The Original Hemp Buds (OR or NY, USA; *N*=1; strain: Electra (H8-1)). THC-positive marijuana samples from the USA-Mexico border were obtained from U.S. Customs and Border Protection (N=4 from different seizures (10-A1, 12-A7, 16-B1, 21-A16)). Chile marijuana DNA extracts were received from the Policia de Investigaciones in southern Chile (*N*=2 from separate cases (35,41)). A Chilean medical marijuana DNA extract was provided by collaborators in Chile (N=1; strain: London Cheese (MedMJ10)).

### DNA extraction and quantification

Total genomic and organelle DNA was extracted using the DNeasy® Plant Mini kit (QIAGEN, Hilden, Germany) (*N*=8 samples) or nexttec™ 1-Step DNA Isolation Kit for Plants (nexttec, Hilgertshausen, Germany) (*N*=3 samples) according to the manufacturers’ protocols. Extraction from USA-Mexico marijuana was performed on-site at CBP, and extraction from hemp was performed at Sam Houston State University. Chilean samples were provided by collaborators in the form of DNA extracts. Chloroplast DNA was quantified using a real-time PCR method reported by Houston et al. (Houston et al., 2018a, b).

### Target enrichment

Seven polymorphic “hotspot” regions in the chloroplast of *C. sativa* (*trnK-matK-trnK*, *rps16*, *trnS-trnG*, *ycf3*, *accD-psaI*, *clpP*, and *rpl32-trnL*) were amplified in single PCR (Roman et al. 2019). Primers were designed using Primer3 (Koressaar and Remm 2007) and checked for specificity using the Primer-BLAST tool (NCBI). The optimal annealing temperature for each primer set was determined by gradient Polymerase Chain Reaction (PCR) as previously described (Roman et al. 2019). PCR was carried out on a Veriti™ 96-well Thermal Cycler (Thermo Fisher Scientific, Waltham, MA) using the TaKaRa LA PCR™ Kit Ver.2.1 (TaKaRa Bio Inc., Kusatsu, Shiga, Japan). Reactions consisted of 0.25 μL TaKaRa LA Taq polymerase, 2.5 μL 10X LA PCR Buffer II (Mg^2+^ free), 2.5 μL 25 μM MgCl_2_, 4 μL dNTP mix, 2.5 μL 2 μM primer mix (Table 1), 4 μL template DNA (20 pg/μL), and 9.25 μL water. Cycling conditions consisted of a 2 min initial denaturation at 94 °C; followed by 30 cycles of 98 °C for 10 s, the optimal annealing temperature (Table 1) for 1 min, and 68 °C for 2 min; and a 10 min final extension at 72 °C. A negative template control (NTC) was included.

**Table 1:** Primer sequences and optimal annealing temperatures for single PCR reactions

Region	Forward primer	Reverse primer	Product size (bp)	Ta (°C)
*trnK-matK-trnK*	ACGAGCCAAAGTTTTAACACAGG	TCGGCTTTTAAGTGCGGCTA	2111	69
*rps16*	AGAAAAGGGTGTAGACGAACG	TCGTTTCTCGGAGGCAAGAAT	1398	66
*trnS-trnG*	TCTAATGATCCGGGGCGTAA	TGCATTCAAAACGACCTGC	1668	66
*ycf3*	ACGGCTCAGCAGTCAAGTTC	TTCGAAATTCATGAAAGGCCCC	2095	68
*accD-psaI*	GGCTGTTCAAACAGGTACAGG	TGCCGGAAATACTAAGCCCA	1424	68
*clpP*	TAAATTCCCCTGTCGGTGCC	ATGCCTATTGGTGTTCCAAAAGTA	1984	66
*rpl32-trnL*	GGAAAAACCCACATACGGCG	TAACACTCGGCGCGGTTATT	1964	69

Following amplification, samples were quantified using the Qubit™ dsDNA HS Assay Kit™ (Invitrogen, Carlsbad, CA) on a Qubit™ 2.0 fluorometer (Invitrogen). The seven PCR targets were then diluted and pooled to a final concentration of 1 ng/μL. A 25 μL aliquot of the mixed PCR products was moved to a new tube and incubated with 2 μL exonuclease I (10 U/μL, Invitrogen) at 35 °C for 72 min and 75 °C for 15 min to remove excess primers.

### Library preparation and sequencing

The Nextera XT DNA Library Preparation Kit (Illumina, San Diego, CA) was used for tagmentation and indexing of libraries. A 1 ng input of amplified, cleaned DNA was used (1 μL), and libraries were prepared according to the manufacturer’s instructions with the exception that 15 cycles of PCR were used for indexing instead of 12 cycles to ensure adequate library quantity (Illumina 2018). Sample libraries were checked on the 2100 Bioanalyzer (Agilent, Santa Clara, CA) using the High Sensitivity DNA kit (Agilent). Following manual normalization, all 14 libraries and the NTC were pooled in equal amounts and denatured according to the manufacturer’s instructions, resulting in a 15 pM library (Illumina 2019). A denatured PhiX control (20 pM) was spiked in at 5% volume. Sequencing on the MiSeq FGx® (Verogen, San Diego, CA) was performed using the MiSeq FGx® Reagent Micro Kit (Verogen).

### Data analysis

Data analysis was performed using the Miseq Control Software v1.4.0.0, MiSeq Reporter v2.5.1.3, and Real-Time Analysis v.1.18.54.0 software installed on the instrument. Variants were reported at a minimum coverage of 10 reads, Q30 variant score, and a minimum variant percentage of 20%. Sequences were compared to a *C. sativa* chloroplast reference genome (Yoruba Nigeria cultivar, GenBank accession NC_027223.1). Variants to the reference genome were reported in a variant call file, and .bam files were viewed in Integrative Genomics Viewer (*IGV*) 2.8.0 (Robinson et al. 2011).

## Results and discussion

### Sequencing metrics

All samples (*n*=14) were successfully sequenced. Sequencing quality was high, with 81.2% of base calls having a quality score of 30 or higher, which indicates at least 99.9% accuracy at each base. The error rate in the PhiX control was below 3%. The yield was 1.48 Mb, cluster density was 1045 ± 26 K/mm^2^, and 99.33% ± 0.26% of clusters passed the filter. Phasing/prephasing rate was 0.139/0.036. Coverage varied within each of the amplicons. The *trnK-matK-trnK* and *rps16* amplicons consistently had the lowest coverage, and *ycf3* consistently had the highest. The clusters passing filter (PF) and clusters aligned to the reference genome for each sample are shown in Supplemental Table 1.

Due to the homopolymeric nature of many of the repeat units (hSTRs), forward and reverse stutter was observed. However, based on the sequence coverage, confident allele calls were made. Some reads aligned to portions of the genome outside of the hotspot regions and were not interpreted, and additionally, several reads misaligned within hotspot regions. These misalignments had low read depth and did not affect the interpretation of sequencing results.

### Sequence data

There were 33 known polymorphisms within the “hotspot” amplicons (Roman et al. 2019 and Cheng and Houston, 2021), and 16 more were discovered, bringing the total to 49 polymorphisms. Newly discovered polymorphisms are indicated by asterisks in Tables 2, 3, 4, 5, 6, 7, and 8. Novel polymorphisms and new variants were reported to GenBank (Accession numbers: MW010378–MW010419). The *trnS-trnG* and *rpl32-trnL* hotspots had the most new polymorphisms with 12 and 11, respectively, and *ycf3* had the least with only three. Analysis of a higher number of polymorphic loci is expected to show increased differences between samples from different populations. However, it is important to note that the genetic boundaries between hemp and drug-type marijuana have been extensively blurred due to the increased demand for high cannabidiol (CBD) strains, resulting in marijuana and hemp cross-breading. In addition, the relationship between cannabinoid synthase gene diversity and cannabinoid content is complex and not fully understood (Grassa et al. 2021).

**Table 2:** Genotypes for polymorphisms in the *trnK-matK-trnK* region (2233–4337 bp)

Sample name	trnK-matK-trnK INDEL (GAATAC)	trnK-matK-trnK SNP (C/T)	***SNP***^a^ ***(T/C)***	***SNP***^a^ ***(A/C)***	trnK-matK-trnK STR1 (A)	trnK-matK-trnK STR2 (T)
**Start location (bp)**	2984	3258	3561	3752	3809	4109
Yoruba Nigeria (NC_027223.1)	Absent	C	T	A	11	10
H2-4	Absent	C	T	A	11	10
H3-3	Absent	C	T	A	11	10
H5-4	Present	C	T	A	11	10
NT H5-1	Absent	C	T	A	11	10
NT H5-2	Present	C	T	A	11	10
NT H5-4	Present	C	C	C	15	9
H8-1	Present	C	T	A	11	10
10-A1	Present	C	T	A	11	10
12-A7	Present	C	T	A	11	10
16-B1	Present	C	C	C	13	9
21-A16	Present	C	T	A	11	10
35	Present	C	C	C	15	9
41	Present	C	C	C	14	9
MedMJ10	Present	C	T	A	11	10

Italics indicate polymorphisms not analyzed previously by CE

^a^ Indicates new polymorphisms discovered in this study

**Table 3:** Genotypes for polymorphisms in the *rps16* region (4803–6201 bp)

Sample name	***INDEL***^a^ ***(AAAGTA)***	***trnK-rps16 hSTR (A)***	rps16 SNP1 (A/G)	rps16 SNP2 (A/C)	rps16 hSTR (C)	rps16 SNP3 (G/A)
**Start location (bp)**	4911	5197	5303	5517	5518	6103
Yoruba Nigeria (NC_027223.1)	Absent	9	A	A	12	G
H2-4	Absent	9	A	A	11	G
H3-3	Absent	9	A	A	11	G
H5-4	Absent	10	A	A	11	A
NT H5-1	Absent	9	A	A	11	G
NT H5-2	Absent	10	A	A	11	A
NT H5-4	Present	11	A	A	13	G
H8-1	Absent	10	A	A	11	A
10-A1	Absent	10	A	A	11	A
12-A7	Absent	10	A	A	11	A
16-B1	Present	11	A	A	14	G
21-A16	Absent	10	A	A	11	A
35	Present	11	A	A	13	G
41	Present	11	A	A	14	G
MedMJ10	Absent	10	A	A	11	A

Italics indicate polymorphisms not analyzed previously by CE

^a^ Indicates new polymorphisms discovered in this study

**Table 4:** Genotypes for polymorphisms in the *trnS-trnG* region (8300–9967 bp). Discrepancies between the genotypes obtained by MPS and CE are indicated in parentheses

Sample name	***psbI-trnS hSTR (T)***	***hSTR***^a^ ***(A)***	trnS-trnG SNP1 (T/C)	trnS-trnG SNP2 (A/T)	***hSTR***^a^ ***(T)***	***INDEL***^a^ ***(CAATAT)***	***SNP***^a^ ***(A/T)***	trnS-trnG hSTR1 (Variable)	trnS-trnG hSTR2 (T)	***hSTR***^a^ ***(A)***	trnS-trnG SNP3 (T/A)	***SNP***^a^ ***(A/C)***
**Start location (bp)**	8359	8585	8595	8684	8685	8731	8877	9018	9104	9149	9360	9463
Yoruba Nigeria (NC_027223.1)	7	10	T	A	9	Absent	A	15	11	12	A	A
H2-4	7	10	T	A	9	Absent	A	15	11	12	A	A
H3-3	7	10	T	A	9	Absent	A	15	11	12	A	A
H5-4	8	10	C	T	9	Absent	A	15	11	12	T	A
NT H5-1	7	10	T	A	9	Absent	A	15	11	12	T	A
NT H5-2	8	10	C	T	9	Absent	A	15	11	12	T	A
NT H5-4	8	12	T	A	8	Absent	A	16	12 (CE: 11)	11	T	C
H8-1	8	10	C	T	9	Absent	A	15	11	12	T	A
10-A1	8	10	C	T	9	Absent	A	15	11	12	T	A
12-A7	8	10	C	T	9	Absent	A	15	11	12	T	A
16-B1	8	10	T	A	8	Absent	T	16	12 (CE: 11)	11	T	C
21-A16	8	10	C	T	9	Absent	A	15	11	12	T	A
35	8	10	T	A	8	Present	A	16	12 (CE: 11)	11	T	C
41	8	10	T	A	8	Absent	T	16	12 (CE: 11)	11	T	C
MedMJ10	8	10	C	T	9	Absent	A	15	11	12	T	A

Italics indicate polymorphisms not analyzed previously by CE

^a^ Indicates new polymorphisms discovered in this study

**Table 5:** Genotypes for polymorphisms in the *ycf3* region (43,383–45,478 bp)

Sample name	ycf3 hSTR1 (T)	ycf3 hSTR2 (T)	ycf hSTR3 (A)
**Start location (bp)**	43454	44007	45034
Yoruba Nigeria (NC_027223.1)	11	11	10
H2-4	11	11	10
H3-3	11	11	10
H5-4	10	12	10
NT H5-1	11	11	10
NT H5-2	9	12	10
NT H5-4	9	10	10
H8-1	10	12	10
10-A1	10	12	10
12-A7	10	12	10
16-B1	9	10	10
21-A16	10	12	10
35	9	10	10
41	9	10	10
MedMJ10	10	12	10

**Table 6:** Genotypes for polymorphisms in the *accD-psaI* region (58,173–59,596 bp)

Sample name	accD-psal SNP1 (A/G)	accD-psal SNP3 (A/C)	***SNP***^a^ ***(G/A)***	***SNP***^a^ ***(T/C)***	accD-psal SNP2 (T/G)	accD-psal STR (A)
**Start location (bp)**	58833	58851	58921	58924	58981	59141
Yoruba Nigeria (NC_027223.1)	A	A	G	T	T	10
H2-4	A	A	G	T	T	10
H3-3	A	A	G	T	T	10
H5-4	G	A	G	T	G	11
NT H5-1	A	A	G	T	T	10
NT H5-2	G	A	G	T	G	11
NT H5-4	G	C	G	T	T	10
H8-1	G	A	G	T	G	11
10-A1	G	A	G	T	G	11
12-A7	G	A	G	T	G	11
16-B1	G	C	A	C	T	10
21-A16	G	A	G	T	G	11
35	G	C	A	C	T	10
41	G	C	A	C	T	10
MedMJ10	G	A	G	T	G	11

Italics indicate polymorphisms not analyzed previously by CE

^a^ Indicates new polymorphisms discovered in this study

**Table 7:** Genotypes for polymorphisms in the *clpP* region (70,502–72,486 bp)

Sample name	clpP hSTR1 (A)	clpP hSTR2 (Variable)	clpP hSTR3 (T)	clpP hSTR4 (T)	clpP INDEL (TTCAATTTA)
**Start location (bp)**	70,912	70,981	71,663	72,016	72,028
Yoruba Nigeria (NC_027223.1)	11	TATTT	14	13	Absent
H2-4	11	TATTT	14	12	Absent
H3-3	11	TATTT	14	12	Absent
H5-4	10	TTTT	15	12	Present
NT H5-1	11	TTTT	14	12	Absent
NT H5-2	10	TTTT	15	11	Present
NT H5-4	11	TTTT	12	11	Absent
H8-1	10	TTTT	14	11	Present
10-A1	10	TTTT	15	11	Present
12-A7	10	TTTT	14	11	Present
16-B1	12	TTTT	11	10	Absent
21-A16	10	TTTT	15	11	Present
35	12	TTTT	11	10	Absent
41	12	TTTT	11	10	Absent
MedMJ10	10	TTTT	15	11	Present

**Table 8:** Genotypes for polymorphisms in the *rpl32-trnL* region (112,153–114,100 bp). Discrepancies between the genotypes obtained by MPS and CE are indicated in parentheses

Sample name	***hSTR***^a^ ***(T)***	***hSTR***^a^ ***(A)***	***ndhF-rpl32 INDEL (Variable)***	rpl32-trnL hSTR1 (A)	***SNP***^a^ ***(A/T)***	rpl32-trnL hSTR2 (A)	***hSTR***^a^ ***(A)***	rpl32-trnL SNP (A/C)	rpl32-trnL hSTR3 (Variable)	rpl32-trnL INDEL (TAAAAA)	***SNP***^a^ ***(A/G)***
**Start location (bp)**	112,294	112,551	112,562	112,830	112,900	112,961	113,017	113,044	113,149	113,246	113,459
Yoruba Nigeria (NC_027223.1)	8	12	Absent	12	A	11	10	A	8 (7T+A)	Present	A
H2-4	8	12	Absent	12	A	11	10	A	9( 8T+A)	Absent	A
H3-3	8	12	Absent	12	A	11	10	A	8 (7T+A)	Absent	A
H5-4	8	13	GAATTG+10A	11	A	11	10	C	6 (6A)	Present	A
NT H5-1	8	11	Absent	12	A	12	10	A	6 (2T+4A)	Present	A
NT H5-2	8	13	GAATTG+11A	11	A	11	10	C	6 (6A)	Present	A
NT H5-4	8	11	GAATTG+10A	11	T	11 (CE:12)	11	A	11 (7T+4A)	Present	A
H8-1	8	13	GAATTG+12A	11	A	11	10	C	6 (6A)	Present	A
10-A1	8	13	GAATTG+11A	11	A	11	10	C	6 (T+5A)	Present	A
12-A7	8	13	GAATTG+12A	11	A	11	10	C	6 (6A)	Present	A
16-B1	7	11	GAATTG+10A	11	T	11 (CE:12)	11	A	14 (6T+8A)	Present	G
21-A16	8	13	GAATTG+11A	10	A	11	10	C	6 (6A)	Present	A
35	7	10	GAATTG+10A	11	T	11 (CE:12)	11	A	15 (11T+4A)	Present	G
41	7	10	GAATTG+10A	11	T	11 (CE:12)	11	A	14 (6T+8A)	Present	G
MedMJ10	8	13	GAATTG+11A	11	A	11	10	C	6 (6A)	Present	A

Italics indicate polymorphisms not analyzed previously by CE

^a^ Indicates new polymorphisms discovered in this study

The genotypes at each locus for 14 samples are displayed in Tables 2, 3, 4, 5, 6, 7, and 8. Samples H8-1 (USA hemp) and 12-A7 (USA-Mexico marijuana) were the only two that produced the same haplotype. These two samples were analyzed previously with CE-based methods and also shown to have the same haplotype (Roman et al. 2019; Roman and Houston 2020). Samples 10-A1 (USA-Mexico marijuana) and MedMJ 10 (Chile medical marijuana) were also shown to have the same haplotype in previous studies. However, using MPS, they were distinguished by their sequences at *rpl32-trnL* hSTR3; 10-A1 has a 6-bp allele with the sequence TAAAAA, and MedMJ10 has a 6 bp allele with the sequence AAAAAA. Since they are the same size, these two isoalleles could not be distinguished in previous CE-based studies (Roman et al. 2019).

### Concordance

Previously, 30 polymorphisms within the seven hotspot regions were analyzed in our laboratory using CE-based methods (Roman et al. 2019; Roman and Houston 2020; Cheng and Houston, 2021). The genotypes obtained by MPS are fully concordant with the CE-based genotypes with exceptions at the *trnS-trnG* hSTR2 and *rpl32-trnL* hSTR2 loci. In four samples (NT H5-4, 16-B1, 35, and 41), the sequence genotypes at both loci appeared to be off by 1 bp from the CE-based genotypes (indicated in Tables 4 and 8). The sequence data showed that the CE fragment assays for both of these loci amplified regions containing the locus of interest as well as an additional hSTR locus that was unknown at the time. Variation at this new hSTR locus explains the discrepancy between the CE and sequencing genotypes for all four samples at both loci. Sequence data for the two alleles observed at trnS-trnG hSTR2 were submitted to GenBank (accession numbers: MW010378–79), and these sequences include the newly observed hSTR.

Previously, *rps16* hSTR and *clpP hSTR3* alleles were reported by their bp size due to unclear results using Sanger sequencing (Roman and Houston 2020). However, as expected, the MPS method was able to elucidate the sequences of all alleles (GenBank accessions: *rps16* hSTR (MW010380–83) and *clpP hSTR3* (MW010383–86), and the fragment size and sequence data were determined to be concordant. Additionally, the 11 and 15 alleles at the *rpl32-trnL* hSTR3 locus were unable to be confirmed by Sanger sequencing in the previous study (Roman et al. 2019), but MPS was able to provide sequence data for these alleles (GenBank accessions: MW010387–88).

## Conclusions

The MPS assay developed in this study provided an effective method for genotyping seven chloroplast regions previously shown to be informative for intra-species variation of *C. sativa*. It provided multiple benefits over previous CE-based assays, including simultaneous analysis of all seven regions in multiple samples, higher confidence for haplotype calls, and better discrimination through sequencing more polymorphisms and identifying isoalleles. A preliminary set of 14 samples was sequenced, and a total of 49 polymorphisms were observed, 16 of which have not been previously published. The sequence data were concordant with CE genotypes from previous studies. The high throughput ability of MPS will allow for the creation of a worldwide haplotype database of *C. sativa* samples. Additionally, a comprehensive database is necessary to understand intra-species variation and could aid law enforcement trafficking cases.

## Supplementary Information

**Additional file 1.**

## Data Availability

All data generated or analyzed during this study are included in this published article [and its supplementary information files].

## Abbreviations

CE: Capillary electrophoresis
ISFG: International Society of Forensic Genetics
MPS: Massively parallel sequencing
NGS: Next generation sequencing
NTC: Negative template control
PCR: Polymerase chain reaction
PF: Passing filter
STR: Short tandem repeat
hSTR: Homopolymeric short tandem repeat
