# Supplementary material for: Massively parallel sequencing of Cannabis sativa chloroplast hotspots for forensic typing
Source: J Cannabis Res. 2022 Mar 17;4:13. doi: 10.1186/s42238-022-00123-2 (PMC8928601; doi:10.1186/s42238-022-00123-2)
Supplement: Supplementary file 1 — Additional file 1. [file 42238_2022_123_MOESM1_ESM.docx]

**Supplemental Table 1** Sequencing coverage for each sample

| Sample Name | Cluster PF | Cluster Align |
| --- | --- | --- |
| H2-4 | 354478 | 340754/338903 |
| H3-3 | 421534 | 397646/396664 |
| H5-4 | 499828 | 468880/466048 |
| NT H5-1 | 324939 | 318659/316935 |
| NT H5-2 | 329130 | 316440/314649 |
| NT H5-4 | 162214 | 145299/144912 |
| H8-1 | 430363 | 410957/409288 |
| 10-A1 | 247712 | 234158/233555 |
| 12-A7 | 295382 | 285361/283439 |
| 16-B1 | 259612 | 251007/249592 |
| 21-A16 | 312004 | 300208/298747 |
| 35 | 286421 | 275212/272900 |
| 41 | 345513 | 335350/333284 |
| MedMJ10 | 301630 | 282215/280864 |
| NTC | 18667 | 144/191 |
